# The Antioxidant Content and Protective Effect of Argan Oil and *Syzygium aromaticum* Essential Oil in Hydrogen Peroxide-Induced Biochemical and Histological Changes

**DOI:** 10.3390/ijms19020610

**Published:** 2018-02-18

**Authors:** Meryem BAKOUR, Najoua SOULO, Nawal HAMMAS, Hinde EL FATEMI, Abderrazak ABOULGHAZI, Amal TAROQ, Abdelfattah ABDELLAOUI, Noori AL-WAILI, Badiaa LYOUSSI

**Affiliations:** 1 Laboratory of Physiology Pharmacology and Environmental Health, Department of Biology, Faculty of Sciences DharMehraz, University Sidi Mohamed Ben Abdellah, 30000 Fez, Morocco; meryem.bakour@usmba.ac.ma (M.B.); soulo.najoua1993@gmail.com (N.S.); abdouaboughazi1@gmail.com (A.A.); taroq.amal@gmail.com (A.T.); abdellaouia@yahoo.fr (A.A.); 2 Laboratory of Biomedical and Translational Research, Faculty of Medicine and Pharmacy, University Sidi Mohamed Ben Abdellah, 30000 Fez, Morocco; nawalhammas@gmail.com (N.H); elfatemihinde@gmail.com (H.E.F.); 3 Department of Pathology, University Hospital Hassan II, 30000 Fez, Morocco; 4 New York Medical Care for Nephrology, New York, NY 11418, USA; drnoori6@yahoo.com

**Keywords:** hydrogen peroxide, oxidative stress, Argan oil, *Syzygium aromaticum* essential oil

## Abstract

Oxidative stress is an important etiology of chronic diseases and many studies have shown that natural products might alleviate oxidative stress-induced pathogenesis. The study aims to evaluate the effect of Argan oil and *Syzygium aromaticum* essential oil on hydrogen peroxide (H_2_O_2_)-induced liver, brain and kidney tissue toxicity as well as biochemical changes in wistar rats. The antioxidant content of Argan oil and *Syzygium aromaticum* essential oil was studied with the use of gas chromatography. The animals received daily by gavage, for 21 days, either distilled water, *Syzygium aromaticum* essential oil, Argan oil, H_2_O_2_ alone, H_2_O_2_ and *Syzygium aromaticum* essential oil, or H_2_O_2_ and Argan oil. Blood samples were withdrawn on day 21 for the biochemical blood tests, and the kidney, liver and brain tissue samples were prepared for histopathology examination. The results showed that the content of antioxidant compounds in *Syzygium aromaticum* essential oil is higher than that found in Argan oil. H_2_O_2_ increased level of blood urea, liver enzymes, total cholesterol, Low Density Lipoprotein (LDL-C), Triglycerides (TG) and Very Low Density Lipoprotein (VLDL), and decreased the total protein, albumin and High Density Lipoprotein-cholesterol (HDL-C). There was no significant effect on blood electrolyte or serum creatinine. The histopathology examination demonstrated that H_2_O_2_ induces dilatation in the central vein, inflammation and binucleation in the liver, congestion and hemorrhage in the brain, and congestion in the kidney. The H_2_O_2_-induced histopathological and biochemical changes have been significantly alleviated by *Syzygium aromaticum* essential oil or Argan oil. It is concluded that the Argan oil and especially the mixture of Argan oil with *Syzygium aromaticum* essential oil can reduce the oxidative damage caused by H_2_O_2,_ and this will pave the way to investigate the protective effects of these natural substances in the diseases attributed to the high oxidative stress.

## 1. Introduction

Free radicals are naturally present in the living organism, and they include reactive oxygen species (ROS) and reactive nitrogen species (RNS) [1]. They are produced during metabolism of energy in the cell as a result of the reduction of oxygen with one electron which forms superoxide anion (O_2_^•−^) and with 2 or 3 electrons which forms H_2_O_2_ by the action of enzymes such as oxidases and with production of hydroxyl radical (OH^•^). Furthermore, nitric oxide (NO) with anionic superoxide (O_2_^•−^) gives peroxynitrite (ONOO^−^), which is RNS [2].Pollutions of air and water, toxins, drugs, heavy metals, pesticides, and cigarette smoke play an important role in the production of ROS [3].

When the ROS present in physiological concentration, they play an important role in the maintenance and the functioning of the body, but when their production exceeds the capacity of the cells to trap them, they start a state of oxidation called oxidative stress [4].

When the oxidative stress is moderate, the intervention of endogenous antioxidant systems of the organism can handle the situation to return to the physiological state. However, when oxidative stress becomes chronic, it leads to the appearance of several diseases such as cardiovascular diseases, neurodegenerative diseases, diabetes and cancer [5,6,7,8,9,10,11].

Exogenous antioxidants such as vitamin E and C, phenolics, flavonoids, flavonols, flavones and carotenoids have been found to mitigate the activity of the endogenous antioxidant defense and can protect against diseases that result from oxidative stress [12].

*Argania spinosa (Sapotaceae)* is an endemic tree of south-western Morocco, which gives valuable Argan oil. The extraction of this oil was made by three methods: (i) a traditional method which is very slow and produces oil with an insufficient quality of conservation due to the water added during the process of extraction; (ii) a mechanical press which does not require the addition of water during extraction; and (iii) a solvent extraction method which produces oil with unsatisfactory organoleptic properties compared to the oil extracted by traditional method or by mechanical press. This technique is exclusively used to prepare the oil for cosmetic purposes [13,14].

Argan oil is rich in antioxidant compounds such as caffeic acid, vanillic acid, ferulic acid, resorcinol and catechin [15]. Several studies have shown that Argan oil has beneficial effects against many diseases such as cardiovascular diseases, obesity, cancer, and diabetes [16,17,18,19,20]. 

Essential oil is an odorous product of organic compounds found naturally in aromatic plants, and it is obtained by hydro distillation, steam distillation, and pressing techniques [21,22]. The clove (*Syzygium aromaticum*) is a tree from (*Myrtaceae*) family, and its essential oil has been reported to be one of the strongest essential oil in its antioxidant activity; this is due to the chemical composition especially eugenol.

Several studies have shown that exposure to H_2_O_2_ is an effective technique for inducing oxidative stress in animals. H_2_O_2_ can cause elevation of OH^•^ via the Fenton reaction: Fe^2+^ + H_2_O_2_ → Fe^3+^ + ^•^OH + OH^−^ [23,24,25,26].

In this context, the present study was designed to explore the antioxidant content of Argan oil and *Syzygium aromaticum* essential oil, and to investigate the protective effect of Argan oil administered alone and the effect of the formulation of *Syzygium aromaticum* essential oil emulsified in Argan oil against the harmful toxicity induced by H_2_O_2_. 

## 2. Results

### 2.1. Chemical Composition of Syzygium aromaticum Essential Oil and Argan Oil

*Syzygium aromaticum* essential oil was obtained with a percentage of 12.6% (*w*/*w*). The chemical composition of *Syzygium aromaticum* essential oil obtained with Gas chromatography–mass spectrometry (GC/MS) was represented in (Table 1). The result showed that Eugenol (2-Methoxy-4-(2-propenyl) phenol) is the major constituent of the oil with a percentage of 87.03% followed by Eugenyl acetate (4-Allyl-2-methoxyphenyl acetate) with a percentage of 11.25%. The composition of Argan oil includes Schottenol (159 mg/100 g), Spinasterol (129 mg/100 g), Stigmasta-8,22-dien-3β-ol (12 mg/100 g) and other (27 mg/100 g).

### 2.2. Antioxidant Content and Activity of Syzygium aromaticum Essential Oil and Argan Oil

The results showed that the content of antioxidant compounds in *Syzygium aromaticum* essential oil is higher than that found in Argan oil (Table 2). The Total antioxidant capacity (TAC) of the *Syzygium aromaticum* essential oil is higher than Argan oil. 

### 2.3. Effect of the Interventions on Enzymatic Markers

**Figure 1:**
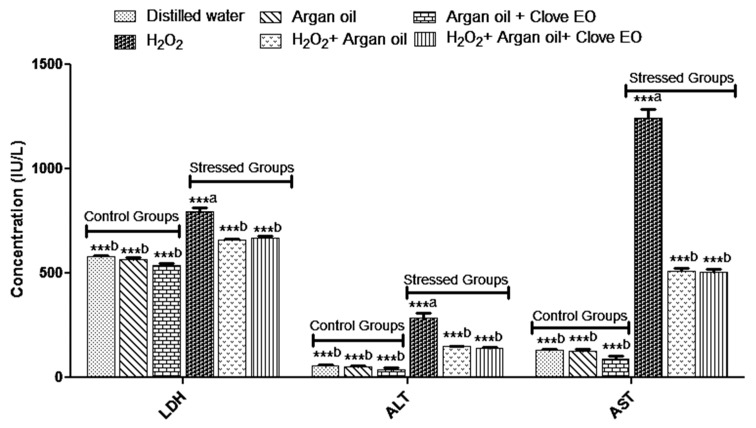
Effect of interventions on Lactate dehydrogenase (LDH), Alanine aminotransferase (ALT) and Aspartate aminotransferase (AST) levels. ^a^: comparison between distilled water group and all groups. ^b^: comparison between H_2_O_2_ group and all groups. *** *p* < 0.05. Data are the means of three replicates (*n* = 3) and presented as mean ± SD.

The result (Figure 1) showed that Argan oil and clove essential oil prepared in Argan oil alleviated the effect of H_2_O_2_ on Lactate dehydrogenase (LDH), Alanine aminotransferase (ALT) and aspartate aminotransferase (AST). H_2_O_2_ increased level of liver enzymes.

### 2.4. Effect of the Interventions on Total Protein and Albumin Levels

**Figure 2:**
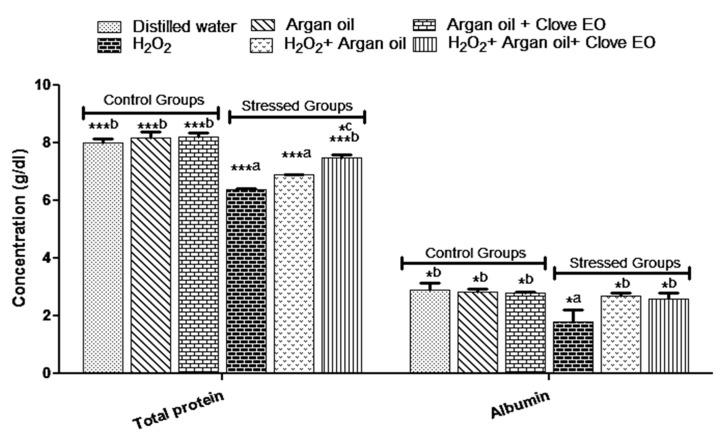
Effect of the interventions on total protein and albumin levels. ^a^: comparison between distilled water group and all groups. ^b^: comparison between H_2_O_2_ group and all groups, ^c^: comparison between H_2_O_2_ + Argan oil and H_2_O_2_ + Argan oil + clove essential oil. * *p* < 0.05, *** *p* < 0.001. Data are the means of three replicates (*n* = 3) and presented as mean ± SD.

The result showed that H_2_O_2_ significantly decreases the total protein and albumin (Figure 2). However, when H_2_O_2_ was used with Argan oil or with Argan oil and clove essential oil, there was no significant change in the total protein or albumin as compared to the water group except for total protein in group that received H_2_O_2_ with Argan oil.

### 2.5. Effect of the Interventions on TC, TG, LDL-C, HDL-C and VLDL Levels

**Figure 3:**
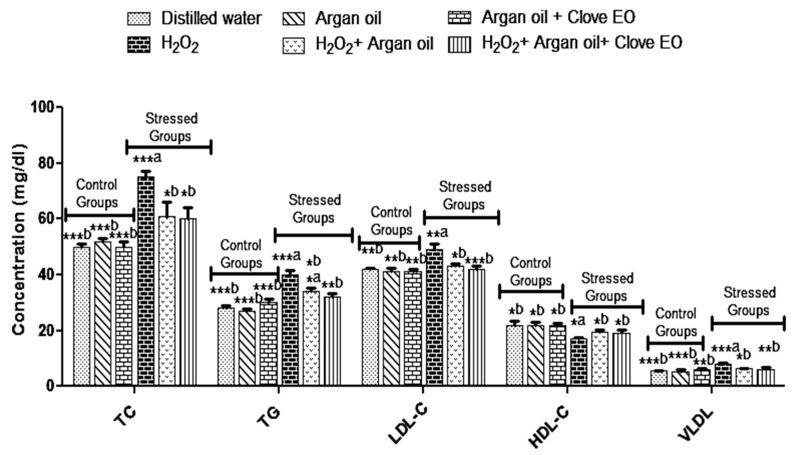
Effect of interventions on TC, TG, LDL-C, HDL-C and VLDL levels. ^a^: comparison between distilled water group and all groups. ^b^: comparison between H_2_O_2_ group and all groups. * *p* < 0.05; ** *p* < 0.01; *** *p* < 0.001. Data are the means of three replicates (*n* = 3) and presented as mean ± SD.

H_2_O_2_ increases the total cholesterol, LDL-C, TG and VLDL and decreases HDL-C, whereas in groups that received Argan oil or Argan oil with essential oil of *Syzygium aromaticum*, there was no significant change in these parameters (Figure 3).

### 2.6. Effect of the Interventions on Serum Electrolytes

H_2_O_2_ alone or with use of argan oil or *Syzygium aromaticum* essential oil with Argan oil did not cause significant changes in the blood electrolytes (Table 3).

### 2.7. Effect of the Interventions on Blood Urea and Creatinine Levels

H_2_O_2_ did not cause a significant change in the plasma creatinine as compared to the control. Blood urea is significantly increased in the group received H_2_O_2_, while in groups received H_2_O_2_ with Argan oil or Argan oil with *Syzygium aromaticum* essential oil, there was no change in the blood urea level (Table 4).

### 2.8. Effect of the Interventions on Organs Weights

Liver and kidney weights and relative weights were significantly increased in the H_2_O_2_ treated group, while brain weight and relative brain weight were significantly decreased (Table 5). The same results were encountered in the group received H_2_O_2_ with Argan oil. However, in the group received H_2_O_2_ with Argan oil with *Syzygium aromaticum* essential oil, there was no changes in the kidney weight and relative kidney weight.

### 2.9. Effects of the Interventions on Histopathological Changes

#### 2.9.1. Brain

**Figure 4:**
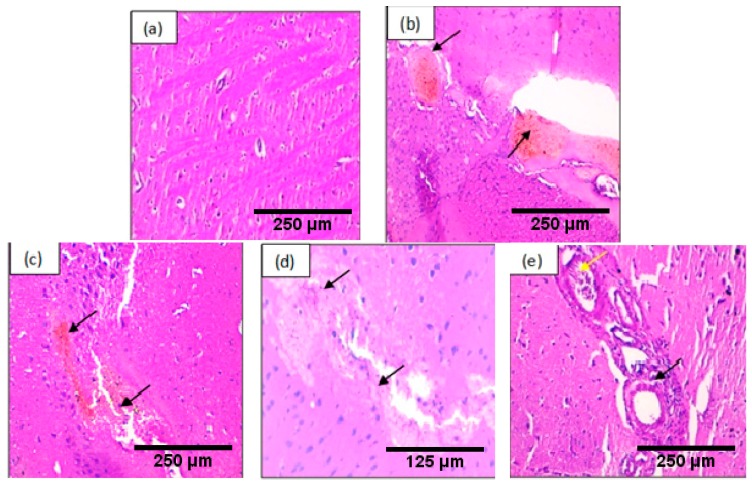
Histopathological evaluation of the brain of the control and stressed groups, the samples were stained with hematoxillin and eosin, the arrows represent pathological changes in tissue: (**a**) control groups: normal tissue ×200; (**b**) H_2_O_2_ group: congestion ×200; (**c**) H_2_O_2_ group: hemorrhage ×200; (**d**) H_2_O_2_ + Argan group: congestion ×400; (**e**): H_2_O_2_ + Argan + *Syzygium aromaticum* essential oil group: the yellow arrow represent congestion and the black arrow represent inflammatory cells infiltration ×200.

Histopathological examination of the brain tissue (Figure 4) showed that H_2_O_2_ induces congestion and hemorrhage. However, it did not induce brain hemorrhage when used along with the Argan oil or *Syzygium aromaticum* essential oil emulsified in Argan oil.

#### 2.9.2. Liver

**Figure 5:**
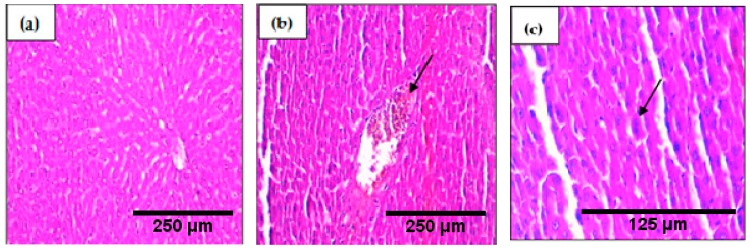

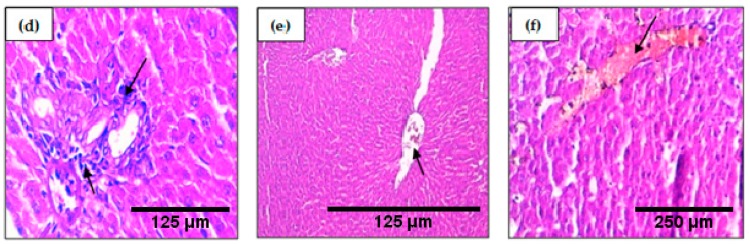
Histopathological evaluation of the liver of the control and stressed groups, samples were stained with hematoxillin and eosin, the arrows represent pathological changes in tissue: (**a**) control groups: normal tissue ×200; (**b**) H_2_O_2_ group: dilatation in the central vein ×200; (**c**) H_2_O_2_ group: binucleation ×400; (**d**) H_2_O_2_ group: inflammation ×400; (**e**) H_2_O_2_ + Argan: dilatation in the central vein ×100; (**f**) H_2_O_2_ + Argan + *Syzygium aromaticum* essential oil group: dilatation in the central vein ×200.

Histopathological examination of the liver tissue (Figure 5) demonstrated that H_2_O_2_ induces dilatation in the central vein, inflammation and binucleation. However, it induced only dilatation in the central vein when it was co-administered with Argan oil or *Syzygium aromaticum* essential oil emulsified in Argan oil.

#### 2.9.3. Kidney

**Figure 6:**
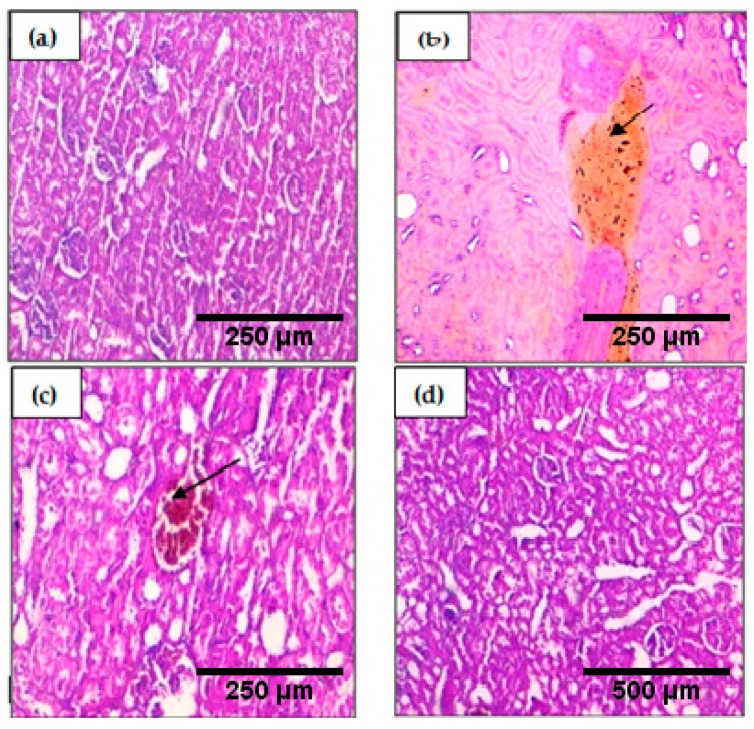
Histopathological evaluation of the kidney of the control and stressed groups, samples were stained with hematoxylin and eosin, the arrows represent pathological changes in tissue: (**a**) control groups: normal tissue ×200; (**b**) H_2_O_2_ group: congestion ×200; (**c**) H_2_O_2_ + Argan group: congestion ×200; (**d**) H_2_O_2_ + Argan + *Syzygium aromaticum* essential oil group: normal tissue ×100.

Histopathological examination of the kidney tissue (Figure 6) showed that H_2_O_2_ induces kidney tissue congestion, however, when it was co-administered with *Syzygium aromaticum* essential oil emulsified in Argan oil, H_2_O_2_ did not cause histopathological change in the kidney tissue.

## 3. Discussion

The results of this study showed that Argan oil and *Syzygium aromaticum* essential oil has a protective effect on H_2_O_2_-induced biochemical changes and histopathological injury in kidney, liver and brain. The results demonstrated that *Syzygium* *aromaticum* essential oil has more antioxidant content than Argan oil. H_2_O_2_ causes significant increase in the lipid parameters, liver enzymes, and blood urea, significant increase in the liver and kidney weight, insignificant increase in the serum creatinine and significant decrease in the total protein and albumin. These H_2_O_2_-induced biochemical changes have been alleviated with use of *Syzygium aromaticum* essential oil or Argan oil.

The chemical composition of *Syzygium aromaticum* essential oil showed that eugenol and eugenol acetate were the main components, that was in agreement with other studies [31,32]. The antioxidant content in *Syzygium aromaticum* essential oil is higher than that found in Argan oil. Therefore *Syzygium aromaticum* essential oil might be more powerful as an antioxidant than Argan oil, and this property most likely due to the chemical composition of *Syzygium aromaticum* essential oil, which is rich in eugenol (87.03%) with a potent antioxidant activity [33,34]. 

The in vivo study demonstrated that H_2_O_2_ given in the drinking water (0.5%) causes significant increase in AST and ALT, decreases total protein and albumin, and an elevation of blood urea levels [35]. Another study showed that administration of H_2_O_2_ (0.1%) in drinking water in rats for 25 weeks induced an increase in the malondialdehyde levels, catalase activity, superoxide dismutase and glutathione peroxidase in organs [36]. 

In the present study daily administration of (1%) of H_2_O_2_ by gavage significantly increased the levels of LDH, ALT, AST, however, rats receiving H_2_O_2_ with Argan oil or *Syzygium aromaticum* essential oil emulsified in Argan oil had lower levels of LDH, ALT and AST in comparison with H_2_O_2_ groups. The increase of ALT and AST is an index of liver damage and alterations of liver function due to the release of these enzymes into the bloodstream from the cytosol [37,38].

The results also showed that H_2_O_2_ causes a decrease in albumin and total protein levels, but in the treated groups there is a total protection against the diminution of albumin in the group which receives the Argan oil alone and in the group, that receives Argan oil with *Syzygium aromaticum* essential oil. However, the protection against the diminution of total protein is better with Argan oil supplemented with *Syzygium aromaticum* essential oil than Argan oil alone. The decrease in albumin levels may be due to inflammation or liver failure [39].

Blood electrolyte (Na^+^, K^+^, Cl^−^) and creatinine levels were not changed significantly during the experiments, but blood urea concentration was significantly increased in the group receiving H_2_O_2_ alone. Blood urea is a waste product of protein metabolism, synthesized in the liver and excreted by the kidney. Therefore, high blood urea could be due to renal damage which was not evident with stable and normal plasma creatinine. Furthermore, the elevated liver enzymes indicate abnormal liver function where low blood urea is expected. Therefore, mechanism of elevated blood urea with the use of H_2_O_2_ needs further experiments. 

Regarding the lipid profile the results showed that H_2_O_2_ causes dyslipidemia with a decrease in the level of HDL-C and an increase in the levels of TC, LDL-C, and VLDL, whereas other groups, which received H_2_O_2_ combined with Argan oil or with Argan oil and *Syzygium aromaticum* essential oil, did not show similar dyslipidemia. It is well known that oxidative stress can induce lipid metabolism disorder and lipid peroxidation and this complication can cause many diseases such as cardiovascular diseases [5].

Regarding the organ weight the results showed that H_2_O_2_ significantly increases the liver and kidney weight and relative weights while it decreases the brain weight and the brain relative weight. This was also observed with the co-administration of H_2_O_2_ with Argan oil. However, co-administration with the *Syzygium aromaticum* essential oil and Argan oil did not affect the relative weight of the kidney. The changes in the weights and relative weights of the organs may be due to histopathological changes caused by H_2_O_2._

Interestingly, the present data showed that H_2_O_2_ induces histopathological changes in the liver, brain and kidney that include dilatation in the central vein, binucleation and inflammation in the liver, congestion in the kidney, and congestion, and hemorrhage in the brain tissue. In the liver, the histopathological changed accompanied by elevation of liver enzymes. The brain is known as the most sensitive organ to oxidative stress because of its high oxygen consumption and low antioxidant content [40]. The results are in agreement with a recent study reporting that oxidative stress causes congestion and cerebral hemorrhage [41]. 

The overproduction of free radicals following the gavage of rats by H_2_O_2_ is most likely the main cause of the histological and biochemical changes, which leads to an imbalance between the oxidant/antioxidant ratio.

In conclusion, the study shows that Argan oil and especially the mixture of Argan oil with *Syzygium aromaticum* essential oil can reduce the oxidative stress that is caused by H_2_O_2_. This protection is obviously due to the bioactive molecules and antioxidants such as eugenol in clove essential oil and vanillic acid, syringic acid, vitamin E and ferulic acid in Argan oil [42,43]. Further studies are needed to identify and characterize the most active materials in Argan oil and *Syzygium aromaticum* essential oil that might be suitable to be tested in clinical setting.

## 4. Materials and Methods

### 4.1. Argan Oil

The virgin Argan oil used in this study was obtained by mechanical press extraction from Agadir city, south west of Morocco, and was preserved at 4 °C in the dark container. In order to investigate the antioxidant effect of this oil, an extraction of phenolics compounds was used. Briefly, 10 g of the Argan oil was dissolved in 5 mL of *n*-hexane then extracted by liquid-liquid extraction with 10 mL of methanol/water (*v*/*v*, 60/40). The aliquot of the methanolic extract was preserved for the antioxidant activity testing [44].

### 4.2. Essential Oil Extraction

A total of 100 g clove was subjected to hydro-distillation for 3 h with 600 mL distilled water using a Clevenger-type apparatus modified: the hydrosol was collected in a separator funnel (1 L) so that the heavy essential oil was decanted to the bottom of the flask and collected. Another funnel of distilled water was used to add water to the flask containing the plant material during boiling. The essential oil obtained was collected and dried over anhydrous sodium sulfate and stored in a refrigerator at 4–5 °C prior to analysis. The yield based on dried weight of the sample was calculated.

### 4.3. Characterization andChemical Composition of Syzygium aromaticum Essential Oil

#### 4.3.1. Gas Chromatography Analysis

The isolated oil was diluted with hexane (dilution ratio 1:10), and 1 mL was sampled for the gas chromatographic analysis. Trace gas chromatograph (GC) (ULTRA S/N 20062969, Thermo Fischer, Villebon-sur-Yvette, France) that is equipped with HP-5MS non polar fused silica capillary column (60 m × 0.32 mm, film thickness 0.25 mm) was used. Operating conditions: oven temperature program from 50 °C (2 min) to 280 °C at 5 °C/min and the final temperature kept for 10 min; 2 “split mode” ratio 1:20; carrier gas Azoth (N), flow rate 1 mL/min; temperature of injector and detector (flame ionization detector) were fixed at 250 °C and 280 °C, respectively.

#### 4.3.2. Gas Chromatography–Mass Spectrometry (GC–MS)

The analysis of the volatile constituents was run on a Thermo Fischer capillary gas chromatograph directly coupled to the mass spectrometer system (model GC ULTRA S/N 20062969; Polaris QS/N 210729), using an HP-5MS non polar fused silica capillary column (60 m × 0.32 mm, 0.25 mm film thickness). The operating condition of GC oven temperature was maintained as: initial temperature 40 °C for 2 min, programmed rate 2 °C/min up to final temperature 260 °C with isotherm for 10 min; and injector temperature 250 °C. The carrier gas was helium, flow rate 1 mL/min. The samples were run in hexane with a dilution ratio of 1:10. The volume of injected specimen was 1mL of diluted oil, splitless injection technique; ionization energy 70 eV, in the electronic ionization mode; ion source temperature 200 °C, scan mass range ofm/z 40–650 and interface line temperature 300 °C. The component identification was made by determination of their retention indices (KI) relative to those of a homologous series of n-alkanes (C_8_–C_20_) (Fluka, Buchs/sg, Buchs, Switzerland) and by matching their recorded mass spectra with those stored in the spectrometer database (NIST MS Library v. 2.0, Gaithersburg, MD, USA) and the bibliography [45].

### 4.4. In Vitro Antioxidant Activities of Argan Oil and Syzygium aromaticum Essential Oil

#### 4.4.1. Total Phenolic Content

The determination of the content of phenolic compounds was made by Folin–Ciocalteau method. Gallic acid was used as a reference. *Syzygium aromaticum* essential oil (50 µL) prepared in ethanol or Argan oil (100 µL) were mixed with 500 µL of Folin–Ciocalteau (0.2 N) reagent and 400 µL of sodium carbonate solution. The reaction mixture was incubated for 2 h in the dark, the absorbance was read at 760 nm, and the tests were made in triplicate [46,47].

#### 4.4.2. Flavones and Flavonols

The content of flavones and flavonols was quantified as follows; 250 µL of *Syzygium aromaticum* essential oil prepared in ethanol or 250 µL of Argan oil were mixed with 250 µL of Alcl_3_ solution, the reaction mixture was incubated for 1 h in the dark, the absorbance was read at 420 nm, the tests were made in triplicate, and quercetine was used as reference [48]. 

#### 4.4.3. Total Flavonoids Content

To analyze the content of total flavonoids, 100 µL of *Syzygium aromaticum* essential oil prepared in ethanol or 100 µL of Argan oil were mixed with sodium nitrite (5%) and 150 µL of Alcl_3_ solution 10%, 200 µL of NaOH (1%) 1M was added after 5 min, absorbance of the reaction mixture was measured at 510 nm, the tests were made in triplicate, and quercetine was used as reference [49].

#### 4.4.4. Total Antioxidant Capacity (TAC)

The antioxidant capacity was evaluated by the phosphomolybdenum method. *Syzygium aromaticum* essential oil prepared in ethanol (25 µL) or Argan oil (25 µL) were mixed with 1 mL of reagent solution (6 M sulfuric acid, 28 mM sodium phosphate and 4 mM ammonium molybdate). After 90 min of incubation in a water bath at 95 °C the absorbance of the solution was measured at 695 nm against blank, the tests were made in triplicate, and quercetine was used as reference [50].

### 4.5. Experimental Animals

Thirty-six wistar rats (body weight 200 ± 20.18 g) were used for the experiment. The animals were housed in a standard environmental condition (25 ± 1 °C, 55 ± 5% humidity and 12 h/12 h light/dark cycle) and fed with rodent rats and free access to water. Experiments were conducted in accordance with the internationally accepted standard guidelines for the use of animals, and the protocol was approved by the institutional committee on animal care following the French Technical Specifications for the Production, Care and Use of the Laboratory and approval from the Ethical committee at Faculty of Sciences, Fez, Morocco was obtained.

### 4.6. Experimental Design

To evaluate the protective effect of Argan oil or *Syzygium aromaticum* essential oil prepared in Argan oil, the rats were randomly divided into six groups: Group 1: received (10 mL/kg/bw) of distilled water, Group 2: received *Syzygium aromaticum* essential oil prepared in Argan oil (100 mg/kg/bw), Group 3: received Argan oil (10 mL/kg/bw), group 4: received 1% of H_2_O_2_ (10 mL/kg/bw) and (10 mL/kg/bw) of distilled water, Group 5: received 1% of H_2_O_2_ (10 mL/kg/bw) and *Syzygium aromaticum* essential oil prepared in Argan oil (100 mg/kg/bw), and Group 6: received H_2_O_2_ (10 mL/kg/bw) and Argan oil (10 mL/kg/bw). The interventions were delivered daily by gavage for 21 days. Blood sample was collected from each rat on day 21 and body weight was measured. The kidney, brain, and liver of each rat were removed, weighted and were immediately fixed in formalin solution at (10%). The dose of Argan oil and *Syzygium aromaticum* essential oil were similar to the doses used elsewhere in rats [51,52].

### 4.7. Blood Analysis

After 3 weeks of treatment blood samples are withdrawn from each rat’s heart under anesthesia for analysis of lactate dehydrogenase (LDH); aspartate aminotransferase (AST); alanine transaminase (ALT); chloride; sodium; potassium; total cholesterol; triglycerides (TG); low density lipoprotein (LDL-C); high density lipoprotein (HDL-C); very low-density lipoprotein (VLDL); creatinine; blood urea; albumin; and total protein.

### 4.8. Histopathological Study

The study was conducted at Pathology Laboratory, University Hospital of Fez. After fixing the organs in the formalin solution (10%) for 48 h, the tissue samples were dehydrated in a series of increasing concentration of ethanol and clarified in toluene, then included in the paraffin. Sections of (5–6 mm) were prepared using a rotating microtome and stained with hematoxylin and eosinfor observation under light microscope.

### 4.9. Statistical Analysis

Statistical analysis was carried out using GraphPad Software (San Diego, CA, USA) and data were represented as mean ± SD.ANOVA was performed and followed by Tukey’s multiple comparison tests. Student *t*-test was used to compare between two means. Throughout the analysis, *p* < 0.05 was considered significant.

## Figures and Tables

**Table 1:** Constituents of *Syzygium aromaticum* Essential Oil and their Relative Percentages of Total Chromatogram Area and Kovats Index.

Compounds	Kovats Index	Area (%)	Chemical Formula	Kovats Index (Literature)
Eugenol	1353.00	87.03	C_10_H_12_O_2_	1327.70 [27]
β-Caryophyllene	1428.00	0.69	C_15_H_24_	1433.90 [28]
Eugenyl acetate	1538.00	11.25	C_12_H_14_O_3_	1524.00 [29]
Caryophyllene oxide	1689.00	<0.10	C_15_H_24_O	1606.00 [30]

**Table 2:** Phenolics, Flavones/Flavonols, Flavonoids and Total antioxidant capacity (TAC) Content.

Sample	Phenolics ^1^	Flavones and Flavonols ^2^	Flavonoids ^2^	TAC ^3^
Argan oil (mg Eq/100 g)	41.28 ± 0.40 *	1.80 ± 0.07 *	8.31 ± 1.06 *	90.90 ± 4.53 *
*Syzygium aromaticum* essential oil (mg Eq/100 g)	165.52 ± 9.71	29.60 ± 1.02	44.08 ± 5.34	3235.50 ± 237.40

^1^ equivalent of gallic acid; ^2^ equivalent of quercetin; ^3^ equivalent of ascorbic acid. Data are the mean of three replicates (*n* = 3) and presented as mean ± SD. * Significant as compared to *Syzygium aromaticum* essential oil (*p* < 0.001).

**Table 3:** Effect of Argan Oil and *Syzygium aromaticum* (clove) Essential Oil on Plasma Electrolytes levels.

Minerals	Distilled Water	Argan Oil	Argan Oil + Clove Essential Oil	H_2_O_2_	H_2_O_2_ + Argan Oil	H_2_O_2_ + Argan Oil + Clove Essential Oil
Sodium (mmol/L)	140 ± 30	139 ± 2.1	138 ± 2	143 ± 2	142 ± 1.5	145 ± 2.1
Potassium (mmol/L)	6 ± 1	5.8 ± 0.8	5.6 ± 1.4	6.3 ± 1.2	5.6 ± 1.7	6 ± 0.9
Chloride (mmol/L)	103 ± 1.2	105 ± 3	100 ± 4	106 ± 2.5	102 ± 2.3	102 ± 2

Data are the means of three replicates (*n* = 3) and presented as mean ± SD.

**Table 4:** Effect of the Interventions on Blood Urea and Creatinine Levels.

Renal Markers	Distilled Water	Argan Oil	Argan Oil + Clove Essential	H_2_O_2_	H_2_O_2_ + Argan Oil	H_2_O_2_ + Argan Oil + Clove Essential Oil
Creatinine (mg/dL)	0.7 ± 0.05	0.6 ± 0.03	0.7 ± 0.03	0.75 ± 0.2	0.6 ± 0.04	0.6 ± 0.08
Urea (mg/dL)	24 ± 1 **^,b^	22 ± 0.5 ***^,b^	23 ± 1.5 ***^,b^	30 ± 0.2 **^,a^	22 ± 1.5 ***^,b^	21 ± 0.9 ***^,b^

^a^: comparison between distilled water group and all groups. ^b^: comparison between H_2_O_2_ group and all groups. ** *p* < 0.01; *** *p* < 0.001. Data are the means of three replicates (*n* = 3) and presented as mean ± SD.

**Table 5:** Effect of the Interventions in the Organs Weights.

Parameters	Distilled Water	Argan Oil	Argan Oil + Clove EO	H_2_O_2_	H_2_O_2_ + Argan Oil	H_2_O_2_ + ArganOil + Clove Essential Oil
Body weight (g)	190 ± 10	198 ± 3	192.5 ± 3.53	181 ± 2	198.5 ± 1.5	203 ± 5
Brain weight (g)	1.87 ± 0.1 ***^,b^	1.92 ± 0.12 ***^,b^	1.8 ± 0.15 ***^,b^	1.29 ± 0.11 ***^,a^	1.62 ± 0.04 ***^,a,^ ***^,b^	1.71 ± 0.09 ***^,a,^ ***^,b,^ *^,c^
Liver weight (g)	6.45 ± 0.04 ***^,b^	6.4 ± 0.1 ***^,b^	6.32 ± 0.1 ***^,b^	9.18 ± 0.11 ***^,a^	6.78 ± 0.05 **^,a,^ ***^,b^	6.65 ± 0.06 *^,a,^ ***^,b^
Kidney weight (g)	0.75 ± 0.04 ***^,b^	0.76 ± 0.02 ***^,b^	0.735 ± 0.01 ***^,b^	1.195 ± 0.01 ***^,a^	0.975 ± 0.02 ***^,a^	0.8 ± 0.02 ***^,b,^ **^,c^
Brain relative weight (g/100 g BW)	0.984 ± 0.05 ***^,b^	0.969 ± 0.06 ***^,b^	0.935 ± 0.077 ***^,b^	0.71 ± 0.06 ***^,a^	0.816 ± 0.02 ***^,a,^ ***^,b^	0.842 ± 0.04 ***^,a,^ ***^,b,^ *^,c^
Liver relative weight (g/100 g BW)	3.394 ± 0.02 ***^,b^	3.383 ± 0.06 ***^,b^	3.283 ± 0.05 ***^,b^	5.07 ± 0.06 ***^,a^	3.41 ± 0.025 **^,a,^ ***^,b^	3.27 ± 0.029 *^,a,^ ***^,b^
Kidney relative weight (g/100 g BW)	0.394 ± 0.02 ***^,b^	0.383 ± 0.01 ***^,b^	0.381 ± 0.05 ***^,b^	0.660 ± 0.05 ***^,b^	0.491 ± 0.01 ***^,a^	0.394±0.009 ***^,b,^ **^,c^

^a^: comparison between distilled water group and all groups. ^b^: comparison between H_2_O_2_ group and all groups, ^c^: comparison between H_2_O_2_ + Argan oil and H_2_O_2_ + Argan oil + clove essential oil. * *p* < 0.05; ** *p* < 0.01; *** *p* < 0.001. Data are the means of three replicates (*n* = 3) and presented as mean ± SD.

## Abbreviations

ROS	Reactive oxygen species
RNS	reactive nitrogen species
GC/MS	Gas chromatography–mass spectrometry
DPPH	1,1-diphenyl-2-picrylhydrazyl
TAC	Total antioxidant capacity
LDH	Lactate dehydrogenase
ALT	Alanine aminotransferase
AST	Aspartate aminotransferase
TC	Total cholesterol
TG	Triglycerides
LDL-C	Low Density Lipoprotein
HDL-C	High Density Lipoprotein-cholesterol
VLDL	Very Low Density Lipoprotein
BHT	Butylated hydroxytoluene
H & E	hematoxylin and eosin
